# Robotic surgery of the liver: Italian experience and review of the literature

**DOI:** 10.3332/ecancer.2013.358

**Published:** 2013-09-26

**Authors:** P Reggiani, B Antonelli, G Rossi

**Affiliations:** Division of General Surgery and Liver Transplantation, IRCCS Fondazione Ca’ Granda Ospedale Maggiore Policlinico di Milano, 20122, Italy

**Keywords:** robotic surgery, liver, minimally invasive surgery

## Abstract

Robotic liver resection is a new promising minimally invasive surgical technique not yet validated by level I evidence. During recent years, the application of the laparoscopic approach to liver resection has grown less than other abdominal specialties due to the intrinsic limitations of laparoscopic instruments. Robotics can overcome these limitations above all for complex operations. A review of the literature on major hepatic surgery was conducted on PubMed using selected keywords. Two hundred and thirty-five patients in 17 series were analysed and outcomes such as operative time, estimated blood loss, length of hospital stay, complications, conversion rate, and costs were described. The most commonly performed procedures were wedge resection and segmentectomy, but the predominance of major hepatectomies performed with robotic surgery is likely due to the superior control achieved by the robotic system.

The conversion and complication rates were 4.2% and 13.4%, respectively. Intracavitary fluid collections and bile leaks were the most frequently occurring morbidities. The mean operation time was 285 min. The mean intraoperative blood loss was 50–280 mL. The mean postoperative hospital stay was four to seven days. Overall survival and long-term outcomes were not reported. Robotic liver surgery in Italy has become a clinical reality that is gaining increasing acceptance; a survey was carried out on robotic surgery, which showed that it is perceived as a significant advantage for operators and a consistent gain for the patient. More than 100 robotic hepatic resections have been performed in Italy where important robotic training schools are active.

Robotic liver surgery is feasible and safe in trained and experienced hands. Further evaluation is required to assess the improvement in outcomes and long-term oncologic follow-up.

## Background

Twenty-five years have passed since the first successful minimally invasive surgical procedure [1] and laparoscopic general surgery has grown exponentially with particular regard to certain pathologies and now, besides cholecystectomy [2], it is considered to be the gold standard for surgery of the oesophagogastric junction, adrenal glands, distal pancreas, and spleen [3–9].

The expanding role of laparoscopic surgery is closely associated with technological advances, including the advent of flexible fibreoptic instruments and improved laparoscopic haemostatic devices such as clips, endoscopic staplers, and energy-induced forceps (electrical based and ultrasonic based). The most recent innovation in this field has been robotic-assisted technology.

Multiple large study series have clearly demonstrated superior outcomes with laparoscopic versus conventional open surgery; the benefits of laparoscopy include decreased postoperative pain, morbidity, and length of hospital stay (LOS), improved cosmesis, and overall cost-effectiveness [4, 5, 8, 10, 11]. Nevertheless, the operative complexity that can be achieved with this kind of minimally invasive surgery has slowed the broad adoption of laparoscopy especially in the most challenging hepatobiliary surgery due to the complex vascular and biliary anatomy of the liver, propensity for bleeding, parenchymal friability, and extremely difficult surgical exposure. Considering the limitations of the laparoscopic technique, it is understandable that implementation of laparoscopy in liver surgery has been slow and isolated to high-volume tertiary care centres. Is the robotic technique the answer to overcoming these limitations?

At the end of 2008, a consensus conference on laparoscopic liver resection (LLR) was held in Louisville, Kentucky [12]. During this conference, the criteria that are best suited for this kind of surgery were defined, including lesions that are solitary, 5 cm or less, located in peripheral segments 3–6.

The robotic technique allows improvement over conventional laparoscopy as it mimics the surgeon’s hands to the tip of instruments, which have seven degrees of freedom and can articulate up to 90°. The EndoWrist technology eliminates the fulcrum effect and the surgeon’s natural hand tremors and provides high stereoscopic definition, a steady view and movement scaling into micromotions. The console allows the surgeon to operate from a seated and ergonomic position, with eyes and hands positioned in line with the instruments. The articulated instruments allow operation with just a shorter learning curve than for complex laparoscopic procedures (involving dissection or reconstruction) [13–16] 

We summarise the major issues of reported robotic liver resections (RLRs), with emphasis on clinical outcomes, reporting data on the Italian activity of robotic resective surgery of the liver.

The first operation of general surgery reported in the literature is a robotic-assisted cholecystectomy by Himpens and colleagues [17], and the name of the robot used at that time was ‘Mona’. In March 2002, the first RLR was performed in Italy by Giulianotti [18], who is considered worldwide to be a pioneer of robotic surgery.

With regard to clinical evidence, there are many publications on robotic surgery, the majority of which are nonrandomised prospective studies and case series (level of evidence II). Since 1998, more than 4,000 publications have appeared in various clinical journals, with about half of these in urology, as well as in cardiothoracic, general, gynaecologic surgeries; paediatric surgery, ear nose and throat, and others [2–7, 19–21].

The scientific community has now validated the method, as did the US Food and Drug Administration (FDA), and national and international conferences that are establishing specific associations are becoming more frequent: in September 2012, the Clinical Robotic Surgery Association celebrated its Fourth Annual Congress in Chicago.

Urologists, gynaecologists, and general surgeons are the front runners in the use of robotic surgery.

## Methods

A PubMed search identified a total of 25 publications relevant to robotic liver surgery. Seventeen publications (235 patients reported) that focused on liver resection provided specific patient descriptions and without doubt of data duplication are considered in this review (Table 1).

All of the authors used the Da Vinci robot system (Intuitive, Sunnyvale, California, United States).

## Indications for Robotic Liver Surgery

**Figure 1:**
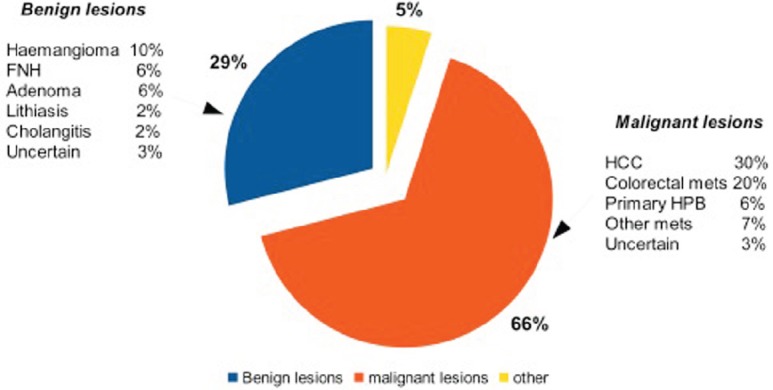
Principal indications for robotic liver surgery.

Initially [21–24], there was a tendency to resect benign lesions, but with an increasing experience, the majority (near 70%) of indications became malignancies. In Figure 1, the main indications of patients analysed in this review are summarised.

The upper limit of tumour size was 5–6 cm in most series, whereas Giulianotti *et al * [22] did not report a size limitation.

The main contraindications to RLR include any of the contraindications for open liver surgery.

## Types of RLRs

The most commonly reported procedure for RLR was wedge resection or segmentectomy (35.2%), followed by left lateral segmentectomy (25.1%) and right hepatectomy (20.6%), as reported in Table 2. Most of the reported cases of right hepatectomy (33/49) were contributed by a single surgeon [22, 25, 37, 38].

The operative time (OR time), defined as the time from skin incision to wound closure was a mean of 285 min (range between 70 and 720 min) with a tendency towards increased OR time observed in series that included major hepatectomies.

Packiam [28] in his comparative study on left lateral sectionectomy (LLS), robotic versus laparoscopic, reported a median OR time of 175 versus 188, respectively.

Intraoperative estimated blood loss (EBL) was reported in six studies [22, 24, 27, 28, 30, 32] and the recorded numerical blood losses ranged from 5 to 2000 mL. Considering the three largest series, [22, 24, 27] the average EBL ranged between 50 and 280 mL. Giulianotti *et al * [22] reported differences in EBL based on different types of pathology: cirrhotic patients had a median EBL of 400 mL (range: 100–1800) higher than noncirrhotic patients. No EBL differences between Lap and robotic LLS also were noted in [28].

Three patients were converted to open surgery for bleeding and three patients for concerns about oncologic integrity (Table 3).

Casciola *et al * [26] reported the experience of posterosuperior hepatic segments robotic resections and conclude that robotic-assisted technique allows carrying out parenchymal-sparing minimally invasive surgery.

## LOS

The duration of postoperative hospital stay was not reported in all the studies and ranged from 2 to 26 days. The median LOS was similar in the publications containing the two largest series, with seven days (range: 2–26) and 5.5 days (range: 3–11). A large variation in LOS between different centres has notably been observed in LLRs. Nguyen *et al * [5] documented average LOS to be 1.9–2.9 days in the United States. Giulianotti *et al * [22] similarly noted a LOS that was two days longer for the resections that were initially done in Italy compared with those later done in the United States.

In the Pittsburgh comparative study [28], on robotic versus laparoscopic left lateral sectionectomies, the LOS was, respectively, four and three days.

## Complications

The overall complication rate was 13.4%, and specific complications are listed in Table 3. Postoperative morbidity included complications specific to the liver (bile leak, transient liver failure, and ascites), surgery-related complications (pleural effusion, wound infection, prolonged ileus, urinary bladder injury, and thoracic empyema), and general complications (transient ischaemic attack and deep vein thrombosis). The most common complication was bile leaks. Out of the four that occurred, two resolved spontaneously, and resolved with drains placed intraoperatively. Two bile leaks required percutaneous drains that were placed postoperatively. Other than bile leaks, the only complication resulting directly from surgical technique was an injury to the urinary bladder. This occurred during the specimen retrieval through a Pfannenstiel incision, and was repaired immediately with an uneventful recovery [24]. 

No complications required reoperation. Ji *et al * [27] observed lower complication rates for their robotic series compared with laparoscopic and open resections (7.8% versus 10% and 12.5%, respectively). Nguyen *et al *reported that the overall complication rate for laparoscopic surgery is 10.5%, which is comparable with the complication rate observed in the robotic series included in this review [5]. No intraoperative mortality occurred in any of the series.

## Oncological Outcomes

Most of the studies considered in our review reported results not differentiated for specific malignancies. As presented by Ho CM in his review [41], we summarise in Table 4 the oncological outcomes if specifically reported for HCC and colorectal liver metastasis (CRLM).

Six patients showed early recurrence. Berber *et al * [30] reported a similar disease-free survival between LLR and RLR.

In the study of Packiam [28], the oncological outcomes for different etiology of six patients with malignancies robotically resected were not reported, the median tumour size in the specimens was 5.5 cm.

## Costs

Two studies analysed costs associated with robotic liver surgery. Berber *et al *reported approximately US$500 extra cost/robotic case compared with the laparoscopic approach [30].

Packiam in his comparative study on lap versus robotic LLS really confirmed the data by Berber. The total surgical supply costs were not significantly different between the robotic and laparoscopic groups (US$5,130 versus US$4,408).

All laparoscopic and robotic instruments composed 79% and 84% of the total supply costs for the average robotic and laparoscopic surgery, respectively.

Staple costs were not significantly different between groups, although there were generally more staple loads used in laparoscopic surgery. While the cost of clips were significantly different between groups, they accounted for <2% of total costs.

The authors concluded that the difference in costs between robotic and lap procedures becomes significantly incremented if indirect costs such as purchase and maintenance of the robot are included.

## The Italian Experience

At the end of 2012, on behalf of the Italian Society of Surgery, for the Updates in Surgery Series a monothematic collection of papers on minimally invasive liver resection (MILR) was edited by Casciola and Calise [42]. In this book, the major issues of laparoscopic and robotic liver surgery are analysed and the results of a survey carried out with the participation of the majority of Italian centres performing MILR are reported. Questionnaires were obtained from 39 centres and a total of 1,677 MILR between 1 January 1995 and 28 February 2012 was reported. The world review of LLRs in [5] enrolled 2,804 liver resections performed in a comparable time period (16 years).

Out of 1677 MILR, 63 robot-assisted hepatic resections are reported and the Centre in Spoleto [26] resulted the most active. In this series, data of two primary robotic Italian centres, Pisa and Grosseto, were not collected. If we add the 47 ‘Italian’ cases of Giulianotti performed during his pioneering activity from 2002 to 2007 when he moved to Chicago, the overall experience counts 110 RLRs. Considering only data published in the papers reviewed, 76 out of 235 are ‘italian’ cases [22, 26, 31]. It is rather difficult to summarise the published Italian experience because Giulianotti in his paper did not split the results and outcomes of the previous Italian experience from the last ‘American’ series. He had conversions to open surgery only in the first half of patients (the Italian era) and no conversions in the second half (overall conversion rate 5.4%, but 8.5% considering the Italian series only). Casciola and Patriti in their series (29 patients) had a conversion rate of 6.9% but a different surgical technique if compared with Giulianotti’s: they widely used parenchymal crushing technique associated to the hepatic inflow control by pedicle clamping (Pringle manoeuvre).

It is also important to report the first robotic right lobe donor hepatectomy performed in Italy on June 2012 by the collaboration of Ismett institute in Palermo and Pisa University Hospital.

Italy ranks first in Europe, together with the more populous Germany, for the number of Da Vinci robotic surgery units available in the country, which in June 2011 were distributed in 46 centres. The most active centres are generally those located in large hospitals with large case studies and various clinical specialties capable of using the equipment.

However, the 46 Robotic centres and 116 dedicated operators represent, in quantitative terms, a small vanguard group if we consider the large number of Italian surgeons, the numerous hospitals and the widespread use of laparoscopy and minimally invasive surgery alone. This activity has already reached high standards: this applies in liver surgery carried out by robotic surgeons in much higher percentages than that of laparoscopic surgeons and traditional open surgery (Table 5). 

Santoro and colleagues [43] reported data of a national survey on robotic surgery in Italy and outlines that on 23 general surgeons who responded to the questionnaire out of 33 suveyed, ten were involved in hepatic surgery.

In Grosseto, a well-known training program is active: the International School of Robotic Surgery founded and directed by Giulianotti and Coratti.

The collaboration between the Grosseto school and other robotic centres around the world produced a new teaching web site: ‘the Clinical Virtual University’, which allows observing live surgery by streaming with comments and interaction with the surgeons in the operative room.

## Discussion

From its initial focus on benign peripheral lesions, minimally invasive surgery has progressively gained broader applications for liver resections [44, 45] (Video 1). Compared with the open approach, laparoscopic liver surgery has several advantages, including decreased postoperative pain, reduced postoperative complications, shorter lengths of stay, and improved cosmesis [5, 46]. Several meta-analyses and case-control studies have demonstrated comparable short- and long-term outcomes for cancer resections [5, 47]. A recent international consensus conference aimed to address and define the role of laparoscopic liver surgery, recognising that it can be done safely in well-selected patients with indicated lesion sites [12]. 

Concerns about laparoscopic surgery are mostly related to the technical complexities of liver resection, risk of intraoperative bleeding, and the difficult learning curve [47, 49].

Robotic-assisted laparoscopic liver surgery has gained attention because it can compensate for the inherent limitations of conventional laparoscopic surgery [50]. The Da Vinci Robot increases the surgeon’s skill and provides more accurate hand–eye coordination, promotes a more ergonomic position at the console, and improves the vision that becomes HD and three-dimensional and also long operations can be performed without fatigue. In some cases, it makes it possible to proceed with surgery that would otherwise be difficult or impossible. Furthermore, it also eliminates the tremor and increases the so-called degree of freedom of operating instruments and their articulated extremities, from four to seven. All this makes the learning process faster, especially for those who already have experience with video-assisted minimally invasive laparoscopic surgery (Video 2)

Considering the types of liver resections performed between robotic and laparoscopic liver surgery, there are mainly differences in percentage of major resections performed. Comparing the extent of RLR to Nguyen *et al *world review to LLRs, there are 45% versus 35% wedges/ segmentectomies, 20% versus 25% left lateral sectionectomies, and 17% versus 38% major hepatectomies [5]. The predominance of major hepatectomies already observed with robotic surgery is likely due to the superior control achieved by the robotic system [49]. In the last few years, the number of minimally invasive complex liver resections has increased, and even segmentectomies of the posterosuperior segments have been successfully performed [26]. Thus, laparoscopic segmental and subsegmental resection of S2, S7, or S8 are an important step towards the fulfilment, even in minimally invasive surgery, of the principles of parenchymal preservation that represents the actual trend in the treatment of both colorectal liver metastases and hepatocellular carcinoma.

If we consider EBL, LOS, and OR time, LLR seems to be superior to RLR, but these outcomes are not precisely defined and clearly described in several studies. While the best way to compare these outcomes should be a randomised controlled trial, this is probably not feasible at this early stage of adoption of robotics.

Rates of conversion to open and postoperative complications are more easily compared; the accepted rates for LLR are 4.1% and 10.5%, respectively. To date, robotics has similar rates of 4.5% and 13.4%. However, a case report by Boggi *et al * [50] illustrated that robotics itself provides significant advantages over laparoscopy during complications. The features of the Da Vinci robot, including the use of three robotic arms by the same operating surgeon (Video 3), use of articulating instruments that can be locked in place as vascular clamps, and ability to perform intracorporeal suturing and tying in difficult locations, are extremely useful in controlling and definitively managing bleeding without necessitating a conversion to open surgery (Video 4).

Results are not yet available to determine long-term oncological outcomes for robotic liver surgery. Most of the reported series have focused on short-term perioperative outcomes [41]. Long-term results and cost-effectiveness are expected to be reported in future studies and are necessary before the advantages and disadvantages of RLR can be conclusively established.

Adequate training is essential to facilitate the use of robotic surgical equipment. Vigano *et al *analysed a 174 patient series over 12 years to show that it takes approximately 60 resections to overcome the learning curve for LLRs [15]. This was based on progressively greater percentages of major hepatectomies performed and malignancies treated. It is possible that the learning curve for robotic resections may be shorter than that of conventional laparoscopic liver surgery. Nevertheless, implementing a new technology such as robot-assisted laparoscopic surgery in a safe and efficient way is demanding. The exponential growth of robotic surgery, however, is not giving the surgical community much time to develop structured training programs for future robotic surgeons. There is a lack of validated training tools for robotic-assisted laparoscopic surgery, and in the near future, further research in this field needs to be performed. With the increasing quality of virtual reality simulators for robotic surgery, it is expected that this training modality will play an important role in training future robotic surgeons, even in terms of costs.

Italy is well placed in the international context, with a limited but significant number of centres, most of which already develops high quantitative and qualitative activities, but high costs justify the use of robotics only for major surgery where the benefit for the patient is more sensitive and the NHS remuneration is higher.

A National Register of robotic surgery is recommended in the near future for verifications, correct information, and validation as is currently done in the United States by the FDA.

## Conclusion

In conclusion, the publications presented in this review show that robotics is a feasible and safe technology that can be used for the resection of hepatic lesions. While outcomes are not till now superior to laparoscopic surgery, we do consider potential to be high for robotics to further expand the application of the minimally invasive approach to liver surgery: the robot could be the future ‘Trojan horse’ carrying in his belly a lot of ‘open’ surgeons through the ‘walls’ of minimally invasive difficult surgery.

## Figures and Tables

**Video 1:**
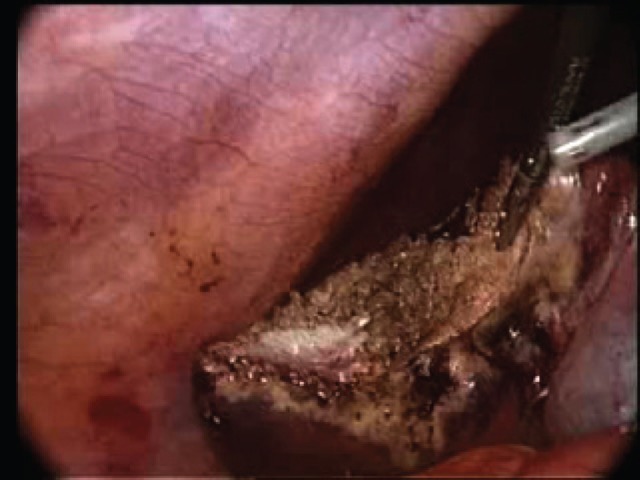
Multiple laparoscopic resections of segment 6 and LLS for metastatic colon malignancy. To view this video click here: http://ecancer.org/journal/7/full/358-Robotic-surgery-of-the-liver-Italian-experience-and-review-of-the-literature.php

**Video 2:**
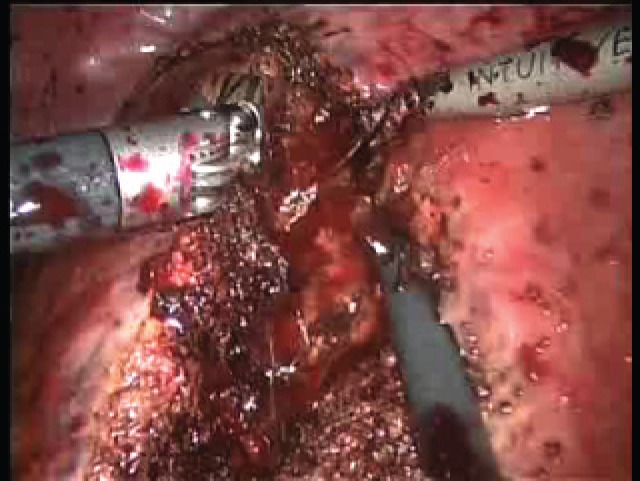
Robotic resection of segment 6 for HCC in cirrhotic patient. To view this video click here: http://ecancer.org/journal/7/full/358-Robotic-surgery-of-the-liver-Italian-experience-and-review-of-the-literature.php

**Video 3:**
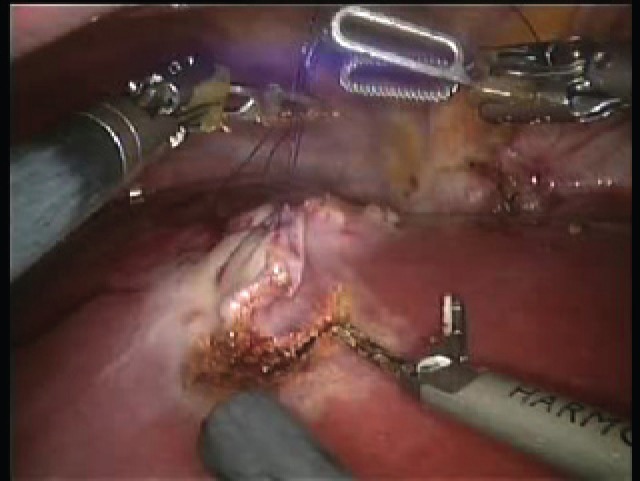
The use of the third arm facilitates the resection lifting the specimen up and opening the surgical field (parachute technique). To view this video click here: http://ecancer.org/journal/7/full/358-Robotic-surgery-of-the-liver-Italian-experience-and-review-of-the-literature.php

**Video 4:**
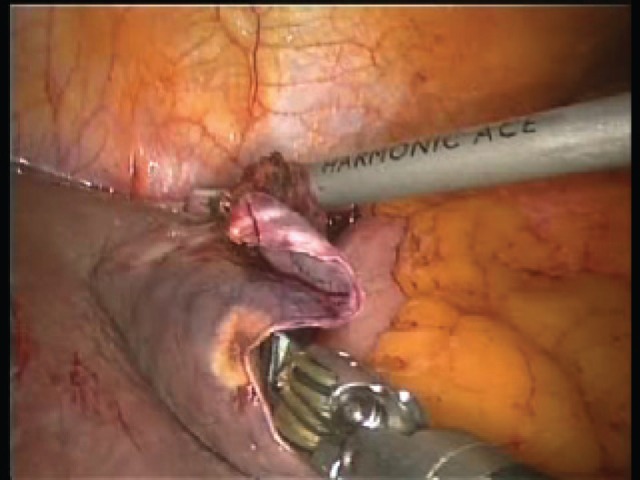
Difficult haemostasis due to the injury of phrenic and left hepatic veins confluence during a segment 2 robotic resection. To view this video click here: http://ecancer.org/journal/7/full/358-Robotic-surgery-of-the-liver-Italian-experience-and-review-of-the-literature.php

**Table 1.** Publications of RLR (listed by number of patients).

References	Years	Journal	No. of Patients Malignant		Benign
Giulianotti *et al *[22]	2011	*Surgery*	70	42	28
Choi *et al *[23]	2012	*Surg Endosc*	30	21	9
Chan *et al *[24]	2011	*J Hepatobiliary Pancreat Sci*	27	21	6
Giulianotti *et al *[25]	2011	*Arch Surg*	24	17	7
Casciola *et al *[26]	2011	*Surg Endosc*	23	19	4
Ji *et al *[27]	2011	*Ann Surg*	13	8	5
Packiam V *et al *[28]	2012	*J Gastrointest Surg*	11	6	5
Lai *et al *[29]	2012	*Int J Surg*	10	9	1
Berber *et al *[30]	2010	*HPB*	9	9	0
Patriti *et al *[31]	2009	*J Hepatobiliary Pancreatic Surg*	6	6	0
Wakabayashi *et al *[32]	2011	*J Hepatobiliary Pancreat Sci*	4	3	1
Vasile *et al *[33]	2008	*Chirurgia (Bucur)*	3	1	2
Panaro *et al *[34]	2011	*JSLS*	1	1	0
Holloway *et al *[35]	2011	*Gynecol Oncol*	1	1	0
Machado *et al *[36]	2009	*Arq Gastroenterol*	1	1	0
Giulianotti *et al *[39]	2012	*Transplant Int*	1	0	1
Ryska *et al *[40]	2006	*Rozhl Chir*	1	0	1

**Table 2.** Types of RLRs performed in the literature reviewed.

Total reported procedures	235
Wedge resection/segmentectomy	81 (35.2%)
LLS	59 (25.1%)
Right hepatectomy	49 (20.6%)
Left hepatectomy	30 (12.5%)
Bisegmentectomy	10 (4.81%)
Right trisectionectomy	2 (0.6%)
Right live donor hepatectomy	1 (0.3%)
Extended right hepatectomy	1 (0.3%)
Pericystectomy	2 (0.6%)

**Table 3.** Complications and conversions for robotic liver surgery (patients = 145).

Deaths	0
Complications	18 (12.8%)
*Liver-related complications*	
Bile leak	4 (2.9%)
Transient liver failure	2 (1.4%)
Ascites	1 (0.7%)
*Surgical-related complications*	
Pleural effusion^a^	3 (2.1%)
Wound infection	2 (1.4%)
Ileus	1 (0.7%)
Urinary bladder injury	1 (0.7%)
Thoracic empyema	1 (0.7%)
*General complications*	
Transient ischaemic attack	2 (1.4%)
Deep vein thrombosis	2 (1.4%)
Diarrhoea	1 (0.7%)
*Conversions*	6 (4.2%)
Open	5 (3.6%)
Hand port	1 (0.7%)

**Table 4.** Oncological outcomes after RLRs for CRLM and HCC.

Author	Pubbl. year	No. patients	Mean tumour size (cm)	Mean follow-up (months)	Oncological Outcome
**CRLM**					
Casciola	2011	14			Two patients died due to tumour progression and three alive with malignant disease.
Giulianotti	2011	11	5.2	36	Two patients with recurrent CRLM at 10 and 20 months were reresected. One patient had bilateral pulmonary metastasis (alive at paper submission). One patient died 12 months after the operation(brain metastasis).
Patriti	2009	6		6.3	One recurrence at seven months.
Choi	2012	4		12	One recurrence at five months.
Berber	2010	4	3.2		One recurrence.
**HCC**					
Choi	2012	13	3.1	12.2	No recurrence.
Casciola	2011	3			One patient died for tumour progression.
Berber	2010	3			One local recurrence six months after resection.
Lai	2012	2	3.8	<1 year	One local recurrence.
Giulianotti	2011	1	6		Alive and no recurrence.

**Table 5.** Oncologic results in comparison with the open/laparoscopic procedure.

10,000 general surgeons–200 hepatic surgeons	(2%)
1,000 laparoscopic surgeons–100 hepatic surgeons	(10%)
23 robotic surgeons–Ten hepatic surgeons	(40%)

## References

[1] Mouret P (1996). How I developed laparoscopic cholecystectomy. Ann Acad Med Singapore.

[2] Polychronidis A (2008). Laparoscopic cholecystectomy in elderly patients. J Gastrointestin Liver Dis.

[3] Atluri P, Woo YJ (2011). Minimally invasive robotic mitral valve surgery. Expert Rev Med Devices.

[4] Martel G, Boushey RP (2006). Laparoscopic colon surgery: past, present and future. Surg Clin North Am.

[5] Nguyen KT, Gamblin TC, Geller DA (2009). World review of laparoscopic liver resection–2,804 patients. Ann Surg.

[6] Levy RM (2010). Laparoscopic and thoracoscopic esophagectomy. Adv Surg.

[7] Morino M (2004). Robot-assisted vs laparoscopic adrenalectomy: a prospective randomized controlled trial. Surg Endosc.

[8] Wilson EB (2009). The evolution of robotic general surgery. Scand J Surg.

[9] Trabulsi EJ (2003). Laparoscopic radical prostatectomy: a review of techniques and results worldwide. Minerva Urol Nefrol.

[10] Galvani C (2006). Laparoscopic adjustable gastric band versus laparoscopic Roux-en-y gastric bypass: ends justify the means?. Surg Endosc.

[11] Bove P (2009). Laparoscopic radical prostatectomy: a review. Int Braz J Urol.

[12] Buell JF (2009). The international position on laparoscopic liver surgery: the Louisville Statement, 2008. Ann Surg.

[13] Wishner JD (1995). Laparoscopic assisted colectomy. The learning curve. Surg Endosc.

[14] Ou YC (2010). Robotic-assisted laparoscopic radical prostatectomy: learning curve of first 100 cases. Int J Urol.

[15] Vigano L (2009). The learning curve in laparoscopic liver resection: improved feasibility and reproducibility. Ann Surg.

[16] Merola S (2002). Comparison of laparoscopic colectomy with and without the aid of a robotic camera holder. Surg Laparosc Endosc Percutan Tech.

[17] Himpens J, Leman G, Cadiere GB (1998). Telesurgical laparoscopic cholecystectomy. Surg Endosc.

[18] Giulianotti PC (2003). Robotics in general surgery: personal experience in a large community hospital. Arch Surg.

[19] Jayne DG (2007). Randomized trial of laparoscopic-assisted resection of colorectal carcinoma: 3-year results of the UK MRC CLASICC Trial Group. J Clin Oncol.

[20] Bivalacqua TJ, Pierorazio PM, Su LM (2009). Open, laparoscopic and robotic radical prostatectomy: optimizing the surgical approach. Surg Oncol.

[21] Idrees K, Bartlett DL (2010). Robotic liver surgery. Surg Clin North Am.

[22] Giulianotti PC (2011). Robotic liver surgery: results for 70 resections. Surgery.

[23] Choi GH (2012). Robotic liver resection: technique and results of 30 consecutive procedures. Surg Endosc.

[24] Chan OC (2011). Robotic hepatobiliary and pancreatic surgery: a cohort study. J Hepatobiliary Pancreat Sci.

[25] Giulianotti PC (2011). Totally robotic right hepatectomy: surgical technique and outcomes. Arch Surg.

[26] Casciola L (2011). Robot-assisted parenchymal-sparing liver surgery including lesions located in the posterosuperior segments. Surg Endosc.

[27] Ji WB (2011). Robotic-assisted laparoscopic anatomic hepatectomy in China: initial experience. Ann Surg.

[28] Packiam V (2012). Minimally invasive liver resection: robotic versus laparoscopic left lateral sectionectomy. J Gastrointest Surg.

[29] Lai EC, Tang CN, Li MK (2012). Robot-assisted laparoscopic hemi-hepatectomy: technique and surgical outcomes. Int J Surg.

[30] Berber E (2010). Robotic versus laparoscopic resection of liver tumours. HPB.

[31] Patriti A (2009). Laparoscopic and robot-assisted one-stage resection of colorectal cancer with synchronous liver metastases: a pilot study. J Hepatobiliary Pancreat Surg.

[32] Wakabayashi G (2011). Our initial experience with robotic hepato-biliary-pancreatic surgery. J Hepatobiliary Pancreat Sci.

[33] Vasile S (2007). The robotic-assisted left lateral hepatic segmentectomy: the next step. Chirurgia (Bucur).

[34] Panaro F (2011). Robotic liver resection as a bridge to liver transplantation. JSLS.

[35] Holloway RW (2011). Robotic-assisted resection of liver and diaphragm recurrent ovarian carcinoma: description of technique. Gynecol Oncol.

[36] Machado MA (2009). First robotic-assisted laparoscopic liver resection in Latin America. Arq Gastroenterol.

[37] Giulianotti PC (2011). Robot assisted laparoscopic extended right hepatectomy with biliary reconstruction. J Laparoendosc Adv Surg Tech A.

[38] Giulianotti PC, Addeo P, Bianco FM (2011). Robotic right hepatectomy for giant hemangioma in a Jehovah’s Witness. J Hepatobiliary Pancreat Sci.

[39] Giulianotti PC (2012). Robot-assisted right lobe donor hepatectomy. Transpl Int.

[40] Ryska M (2012). Manual and robotic laparoscopic liver resection. Two case reviews. Rozhl Chir.

[41] Ho CM (2013). Systematic review of robotic liver resection. Surg Endosc.

[42] Calise F, Casciola L (2013). Minimally Invasive Surgery of the Liver Updates in Surgery.

[43] Santoro E, Pansadoro V (2013). Robotic surgery in Italy national survey (2011). Updates Surg.

[44] Koffron A (2006). Laparoscopic liver surgery: shifting the management of liver tumors. Hepatology.

[45] Wayand W, Woisetschlager R (1993). Laparoscopic resection of liver metastasis. Chirurg.

[46] Nguyen KT (2011). Comparative benefits of laparoscopic vs open hepatic resection: a critical appraisal. Arch Surg.

[47] Mirnezami R (2011). Short- and long-term outcomes after laparoscopic and open hepatic resection: systematic review and meta-analysis. HPB (Oxford).

[48] Buell JF (2008). Experience with more than 500 minimally invasive hepatic procedures. Ann Surg.

[49] Kitisin K (2011). A current update on the evolution of robotic liver surgery. Min Chir.

[50] Boggi U (2009). Robotic suture of a large caval injury caused by endo-GIA stapler malfunction during laparoscopic wedge resection of liver segments VII and VIII en-bloc with the right hepatic vein. Minim Invasive Ther Allied Technol.

